# Rare giant renal artery aneurysm in neurofibromatosis type 1 patient: a case report

**DOI:** 10.1097/MS9.0000000000001329

**Published:** 2023-09-15

**Authors:** Ali Jawad, Zein Alabdin Hannouneh, Jameel Soqia, Zaher Al Nahhas, Adnan Ahmed, Mohamad Ali Nahas

**Affiliations:** a Faculty of Medicine, Damascus University; b Department of Urology, Faculty of Medicine; c Head of Vascular and Endovascular Surgery Division, Al-Assad University Hospital, Damascus University; d Department of Radiology, Damascus Hospital, Damascus; e Faculty of Medicine, Al Andalus University for Medical Sciences, Tartus, Syrian Arab Republic

**Keywords:** case report, nephrectomy, neurofibromatosis type 1, renal artery aneurysm, vasculopathy

## Abstract

**Introduction and importance:**

Neurofibromatosis type 1 (NF1) is a genetic disorder characterised by multiple neurofibromas, café-au-lait spots, and iris hamartomas. The variety of vasculopathies that can occur in NF1 make it difficult for clinicians to accurately follow-up patients. Most cases of vasculopathies are stenotic, and, in few cases, aneurysms may form.

**Case presentation:**

A 35-year-old male presented with extreme left flank pain for the past 2 days. His physical examination revealed whole-body several café-au-lait skin macules, a subcutaneous lesion, and a palpable abdominal mass in the left flank. His laboratory workup was within normal ranges. A multi-slice computed tomography and computed tomography angiogram with contrast outlined a giant left renal artery aneurysm (RAA). A kidney salvage surgery was planned. However, due to ectatic dilatation and large extension of the aneurysm, the affected renal artery branches and renal vein were found unfit for auto-transplantation during the surgical procedure and a total nephrectomy was necessary. Symptoms improved significantly postoperatively and no complications developed.

**Clinical discussion:**

RAA is an uncommon finding in NF1 patients. Diagnosis is often dependent on computed tomography angiogram. Management techniques are conservative, endovascular, or surgical. In few surgical cases, a total nephrectomy may be necessary if auto-transplantation is not feasible.

**Conclusion:**

Despite its rarity, the diagnosis of RAA should be considered in patients with NF1 presenting with flank pain. Moreover, early screening for renal vasculopathies can evade critical surgical outcomes including a total nephrectomy. Hence, the authors recommend a total vascular workup for these patients, consisting of doppler ultrasound and, if necessary, a multi-slice computed tomography with contrast.

## Introduction

Highlights

- Neurofibromatosis type 1 (NF1) is a genetic disorder associated with multiple vasculopathies. Most of which are stenotic, rather than aneurysmal.
- Renal artery aneurysms are uncommon in NF1 patients and are often asymptomatic. They are usually found incidentally on computed tomography. The presence of a symptomatic, large renal aneurysm is rare and challenging for surgeons.
- Management techniques are conservative, endovascular, or surgical. In few surgical cases, a total nephrectomy may be necessary if auto-transplantation is not feasible.
- Early screening for renal vasculopathies in NF1 patients can evade critical surgical outcomes including a total nephrectomy.

Neurofibromatosis type 1 (NF1), as originally described by Von Recklinghausen in 1882^1^, is characterised by multiple non-cancerous tumours originating from peripheral nerve sheaths, called neurofibromas, café-au-lait spots, and iris hamartomas^2^. The cause of this condition is a mutation in the NF1 tumour suppressor gene, which is located on the long arm of chromosome 17 (17q11.2). Almost half of individuals with NF1 mutation have a family history of this condition, while the other half acquire it through de-novo mutations^3^. With an incidence of 1 in 3000 births, this classic form of NF1 is commonly associated with vascular lesions, including coronary artery disease, retinal artery occlusion, peripheral vascular disease, and carotid and renal artery stenosis^4^. Renal artery aneurysm (RAA) is an uncommon vascular disease that is generally detected as an incidental finding during imaging in 0.09% of the general population^5^. Most cases of renal vasculopathies in NF1 are stenotic rather than aneurysmal^6,7^. RAAs usually remain asymptomatic but can cause pain, haematuria, and hypertension as they enlarge^8^. In this report, we present a rare case of an enormous left RAA in a patient with neurofibromatosis who experienced flank pain as the only symptom at presentation. This case report has been reported in line with the SCARE Criteria^9^.

## Case presentation

A 35-year-old male patient presented to our hospital with abdominal pain that transmitted to his left flank for the past two weeks, exacerbated during the last 2 days, and was accompanied by nausea and vomiting. His generalised, persistent abdominal pain was not associated with position or food ingestion. Additionally, it did not improve with symptomatic drugs. The patient had a history of NF1 and underwent surgical excision of a cervical neurofibroma a and fatty mass years ago. The patient had no significant drug history or family history and denied smoking or drinking alcohol.

Upon clinical examination, a pulsatile, mobile mass was palpated in the upper left flank, and multiple skin lesions were observed. Physical examination also revealed whole body, several café-au-lait skin macules and a subcutaneous neurofibroma. His vital signs included a body temperature of 37°C, 96% oxygen saturation, 130/80 mmHg arterial blood pressure, and a heart rate of 90 beats per min. Complete blood count tests, serum creatinine, urinalysis, and serum electrolytes were all within normal limits.

**Figure 1:**
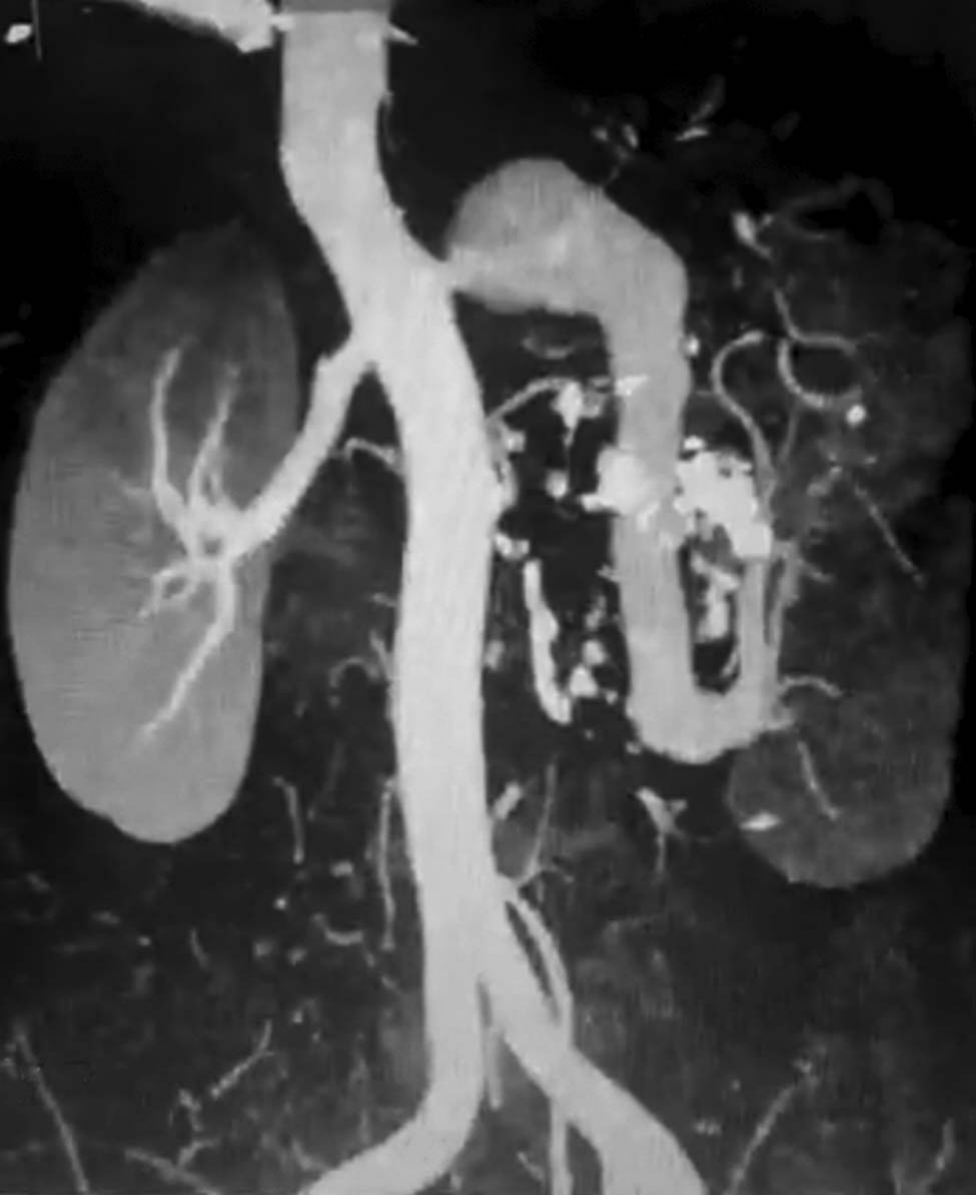
Multi-slice computed tomography angiogram shows a giant and elongated left renal aneurysm. Ectatic dilatation of the upper-lobe renal artery branch is noticed.

**Figure 2:**
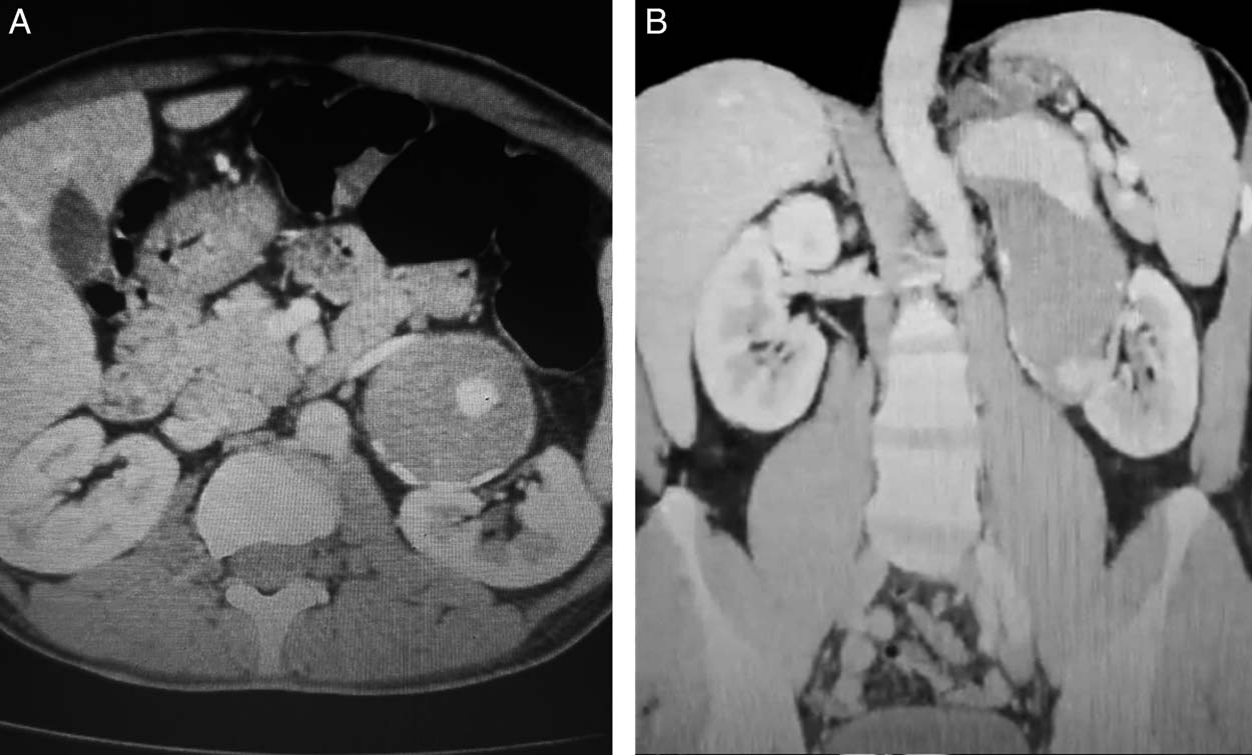
(A) MSCT with contrast (transverse view): a giant left renal aneurysm compared to the aorta, compressing the left renal vein and dislocating the left kidney downward. (B) Multi-slice computed tomography (MSCT) with contrast (coronal view): true left renal artery aneurysm (12.5 ×9 cm), true lumen (4.5 ×3 cm).

The patient underwent an abdominal doppler ultrasound which revealed a large aneurysm of the left renal artery with a patent artery lumen. The kidney was 12 cm in length with normal cortico-mudular differentiation. A multi-slice computed tomography angiogram (CTA) was conducted, which demonstrated a large RAA [Figure 1]. Furthermore, a multi-slice computed tomography with contrast further identified the size of the aneurysmal cavity [Figure 2B] in comparison to the aorta and displacement of the left kidney [Figure 2A]. Radioisotope renography showed a functional left kidney.

The patient was scheduled for surgery with the intention to excise the aneurysm and re-implant the left kidney directly into the left iliac fossa. Consent to perform nephrectomy was obtained in case auto-transplantation was not possible. A left pararectal incision and a retroperitoneal approach was used. The renal vein was found to be elongated, stretched, and compressed by the lesion. During surgical exploration, the aneurysm involved the entirety of the renal arterial trunk and its branches, excluding the possibility of re-implanting the kidney. As a result, a total nephrectomy was conducted. No blood transfusions were required. Repeated lab results included a haemoglobin level of 12.6 g/dl, a haematocrit of 37%, a white blood cell count of 8.82×10^3^/ul, a platelet count of 292×10^3^/ul, and a creatinine level of 0.68 mg/dl. The patient had an uneventful hospital stay and was discharged on the third post-operative day with no complaints.

**Figure 3:**
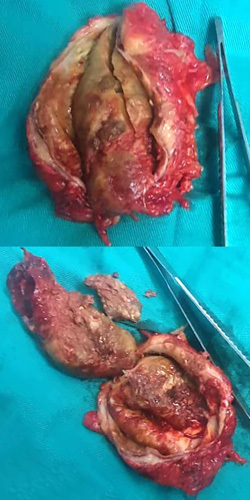
Aneurysmal cyst measuring (12 ×8.5 ×6 cm) with gelatinous haemorrhagic clot (11 ×6 × 5 cm).

Gross examination of the resected left kidney (142 g) revealed a congested pelvicalyceal system. The dissected aneurysm consisted of an open cystic cavity measuring 12 ×8.5 × 6 cm which contained a gelatinous haemorrhagic clot (11 ×6 × 5 cm) [Figure 3]. The aneurysm wall, 1 cm thick, showed fibrosis, calcification, and yellow-coloured tissue. Histological examination of multiple sections from the dissected kidney demonstrated mild focal chronic inflammation with focal haemorrhages. No neoplastic changes were seen. Histological examination of a thick segment of RAA demonstrated fibrosis, calcification, and haemorrhage that replaced most of the arterial wall with missing tunica media and intima. A skin biopsy taken from the patient’s skin lesions was consistent with neurofibroma.

## Discussion

NF1 is a genetic disorder that affects the growth and development of nerve tissue, and it can manifest as various cutaneous, neurological, skeletal, and vascular abnormalities^2^. RAA is uncommon and usually asymptomatic, but it can cause abdominal pain, haematuria, hypertension, and, in rare scenarios, can be complicated by rupture^5^. NF1 may involve stenosis or aneurysm formation at all levels of the renal artery^10^.

The diagnosis of RAA typically involves imaging studies and clinical evaluations. CTA is the most frequent imaging modality used to assess RAAs, followed by non-contrast enhanced computed tomography, magnetic resonance angiography, and ultrasonography^11^. The current vascular surgery guidelines recommend using CTA as the first-line imaging modality in cases of suspected RAA^12^.

The management of RAA depends on several factors, such as the size, location, symptoms, and risk of rupture, as well as the patient’s age, comorbidities, and preferences^5,10^. Some experts suggest that conservative management may be appropriate for asymptomatic small RAA (<2 cm), unless the risk of rupture is elevated (e.g. a female with childbearing potential or who is pregnant)^13^. For larger or symptomatic RAA, surgical or endovascular repair may be indicated. The choice between open surgery and endovascular intervention depends on the technical feasibility, availability of expertise and equipment, and potential complications of each approach^14^.

Previous case reports have described different presentations and management strategies for this condition. Chillura *et al*.^15^ reported a 9-year-old girl with NF1 who had a giant RAA and hypertension. She underwent coil embolization of the aneurysm and was treated with antihypertensive drugs. Uchida *et al* ^16^. reported a 44-year-old man with NF1 who had subarachnoid haemorrhage due to multiple, de-novo aneurysms. He underwent surgical clipping of two aneurysms and endovascular coiling of another one. Aqil *et al*.^17^ reported a 37-year-old woman with NF1 who had bilateral RAA and renal artery stenosis. She underwent angioplasty and stenting of the right renal artery and nephrectomy of the left kidney.

This case report describes a rare association of NF1 with a giant RAA that presented with sudden and severe left flank pain in a 35-year-old male patient. This case report adds to the literature on the association between NF1 and RAA, which is rare and potentially life-threatening. Due to the large size of the RAA (>10 cm), urgent excision of the aneurysm was required due to fear of complications. Kidney re-implantation was infeasible, and a left nephrectomy was performed because the lesion had extended to all the renal branches. This outlines the need for screening NF1 patients for renal vasculopathies to allow for conservative therapy and avoid a total nephrectomy.

The limitations of this case report include the lack of genetic testing for NF1 mutation and the absence of long-term follow-up. Further studies are required to improve our understanding of the pathophysiology, natural history, diagnosis, and treatment of RAA in patients with NF1.

## Conclusion

We present a case of a rare association between NF1 and a symptomatic giant (>10 cm) RAA in a 35-year-old male. The RAA was diagnosed and evaluated with an multi-slice computed tomography and CTA, and surgery was performed accordingly. We believe that there was no indication to endovascular management by stent graft because of the extension and diameter of the aneurysm on one hand and the ectatic dilatation of the branches of the renal artery on the other hand. Surgical repair of the vasculopathy was unfeasible, and a total left nephrectomy was necessary. This case outlines the importance of screening for vascular pathologies in patients with NF1 and keeping RAA as a differential diagnosis, especially in those presenting with flank pain.

## Ethical approval

Given the nature of the article, a case report, no ethical approval was required.

## Consent

Written informed consent was obtained from the patient for publication of this case report and accompanying images. A copy of the written consent is available for review by the Editor-in-Chief of this journal on request.

## Source of funding

Not applicable.

## Author contribution

A.J.: writing—original draft, review and editing. Z.A.H.: writing—original draft, review and editing. J.S.: writing—original draft, review and editing. Z.A.N.: supervision, review and editing, patient’s imaging. A.A.: supervision, review and editing, surgical care and follow-up. M.A.N.: supervision, final review and editing, surgical care and follow-up. All authors read and approved the final manuscript.

## Conflicts of interest disclosure

The authors declare no conflict of interest.

## Research registration unique identifying number (UIN)

Not applicable.

## Guarantor

Mohamad Ali Nahas.

## Provenance and peer review

Not commissioned, externally peer-reviewed.

## Data availability

Not applicable.
